# Patients' perspectives related to ethical issues and risks in precision medicine: a systematic review

**DOI:** 10.3389/fmed.2023.1215663

**Published:** 2023-06-15

**Authors:** Lawko Ahmed, Anastasia Constantinidou, Andreas Chatzittofis

**Affiliations:** ^1^Medical School, University of Cyprus, Nicosia, Cyprus; ^2^Department of Clinical Sciences and Psychiatry, Umeå University, Umeå, Sweden

**Keywords:** patients' perspective, patients' attitude, ethics, precision medicine, personalized medicine

## Abstract

**Background:**

Precision medicine is growing due to technological advancements including next generation sequencing techniques and artificial intelligence. However, with the application of precision medicine many ethical and potential risks may emerge. Although, its benefits and potential harms are relevantly known to professional societies and practitioners, patients' attitudes toward these potential ethical risks are not well-known. The aim of this systematic review was to focus on patients' perspective on ethics and risks that may rise with the application of precision medicine.

**Methods:**

A systematic search was conducted on 4/1/2023 in the database of PubMed, for the period 1/1/2012 to 4/1/2023 identifying 914 articles. After initial screening, only 50 articles were found to be relevant. From these 50 articles, 24 articles were included in this systematic review, 2 articles were excluded as not in English language, 1 was a review, and 23 articles did not include enough relevant qualitative data regarding our research question to be included. All full texts were evaluated following PRISMA guidelines for reporting systematic reviews following the Joanna Briggs Institute criteria.

**Results:**

There were eight main themes emerging from the point of view of the patients regarding ethical concerns and risks of precision medicine: privacy and security of patient data, economic impact on the patients, possible harms of precision medicine including psychosocial harms, risk for discrimination of certain groups, risks in the process of acquiring informed consent, mistrust in the provider and in medical research, issues with the diagnostic accuracy of precision medicine and changes in the doctor-patient relationship.

**Conclusion:**

Ethical issues and potential risks are important for patients in relation to the applications of precision medicine and need to be addressed with patient education, dedicated research and official policies. Further research is needed for validation of the results and awareness of these findings can guide clinicians to understand and address patients concerns in clinical praxis.

## Introduction

Precision medicine, often used interchangeably with the term personalized medicine, is a recently introduced approach to medical care, aiming to provide individualized treatment to patients based on their unique characteristics, including their genes, environment, and lifestyle. This approach has the potential to revolutionize healthcare, with implications across a wide range of medical specialties using different tools including bioinformatics, big data analysis and artificial intelligence/machine learning.

Precision medicine has clinical applications in different medical fields and specialties. Oncology is on the frontier of precision medicine leading to the development of targeted therapies that can selectively kill cancer cells based on their genetic/molecular aberrations (1). In Cardiology, precision medicine has the potential to improve risk stratification and identify patients who are at high risk of developing cardiovascular disease and apply to reduce their risk (2). Even in Psychiatry, precision medicine has the potential to improve the diagnosis and treatment of mental illnesses. For example, in the treatment of depression, genetic testing can identify patients who are likely to respond to specific antidepressants thus reducing the risk of side effects and improving treatment outcomes (3).

Despite the rapid advances, and the obvious prospect of immediate and future applicability of precision medicine in day to day care, important ethical and social issues should be carefully considered and ultimately addressed, to ensure that the benefits of this approach are equitably distributed and that patient rights are protected (4).

Several matters relating to ethical issues and risks in precision medicine are widely known. To start with, concerns have been raised regarding the privacy and security of big data including genetic data, particularly in the context of commercial genetic testing services (5). Commercial genetic testing companies collect large amounts of genetic information from individuals, which can include not only information about an individual's own personal and health data but also information about their relatives. The possibility of this data being misused or mishandled may be significant, with unprecedented consequences for the involved individuals.

In addition, precision medicine has increasing costs, as it often involves advanced diagnostic tests and targeted therapies (5). Thus, some patients may worry about the cost of these treatments and whether they will be covered by insurance. The cost concerns are related also to the risk for unequal access to precision medicine, particularly for marginalized communities who may not have access to genetic testing or targeted therapies. In those cases, the cost of precision medicine may disproportionately affect those who are already disadvantaged, which will lead of widening the gap between less economically developed countries and more economically developed countries. Moreover, the issue of unequal access to precision medicine is not limited to the developing world. Within developed countries, there are also disparities in healthcare access based on factors such as race, ethnicity, and socio-economic status. This means that even if precision medicine becomes more widely available, certain groups may still be left behind (6).

Other ethical issues include challenges in the informed consent process, the potential for stigmatization or discrimination based on genetic information as well as psychological impact and anxiety due to incidental findings (7).

It is important to point out that although the perspectives of patients on precision medicine might overlap with those of doctors, there might be important differences in priorities, concerns, and levels of understanding (7). Understanding the patient's perspective is crucial for ensuring that precision medicine is implemented in an ethical and responsible way and will provide guidance to healthcare professionals and policymakers to address these potential risks and ethical considerations. Although there are studies in specific populations and stakeholders' opinions (7), to our knowledge, there is no systematic review on the patients' perspectives on risks and ethical issues on the implementation of personalization of medicine.

Therefore, the aim of this systematic review was to provide a comprehensive overview and analysis of the perspectives of patients regarding ethical and related issues in precision medicine. This is the first systematic review to focus only on the patients' perspectives in this area.

## Materials and methods

This systematic review was based on the Preferred Reporting Items for Systematic reviews and Meta-Analysis statement (PRISMA) guidelines (8).

### Data sources and searches

The search of the medical literature was conducted in PubMed and was restricted to the last 10 years, including the timeline of 1/1/2012 to 1/1/2023. The research timeframe was chosen considering that although precision medicine applications were evident before, precision medicine expanded greatly in knowledge and applications in the last several years. In addition, previously, different terms were used to describe individualization of therapy, and in the last 10 years, the conceptualization of precision medicine became clearer with the preferred term shifting from personalized medicine to precision medicine (9). Published articles from, January 1st 2012 to January 1^st^ 2023, were collected using a standard search strategy Search query: ((((((((quantitative study) OR qualitative study) OR participant observation) OR focus group) OR interview) OR survey) OR questionnaire) OR patients perspective) AND ((((precision medicine[Title]) OR personalized medicine[Title]) OR personalized medicine[Title]) OR genomic medicine[Title])).

### Study selection

Inclusion criteria included published articles examining the attitudes or views or perspectives of patients toward precision medicine, articles examining patients' and at the same time the public's perspective and articles investigating parents' perspective regarding their children on precision medicine. Exclusion criteria included articles investigating the perspectives or views of the public without the participation of patients. Only articles in the English language were included. Further restrictions were applied to the selected articles based the sections of the PRISMA 2009 checklist (10). However, no further exclusion criteria regarding the sample size, ethnicity, age, and gender were applied.

### Data extraction, quality assessment, and analysis

**Figure 1:**
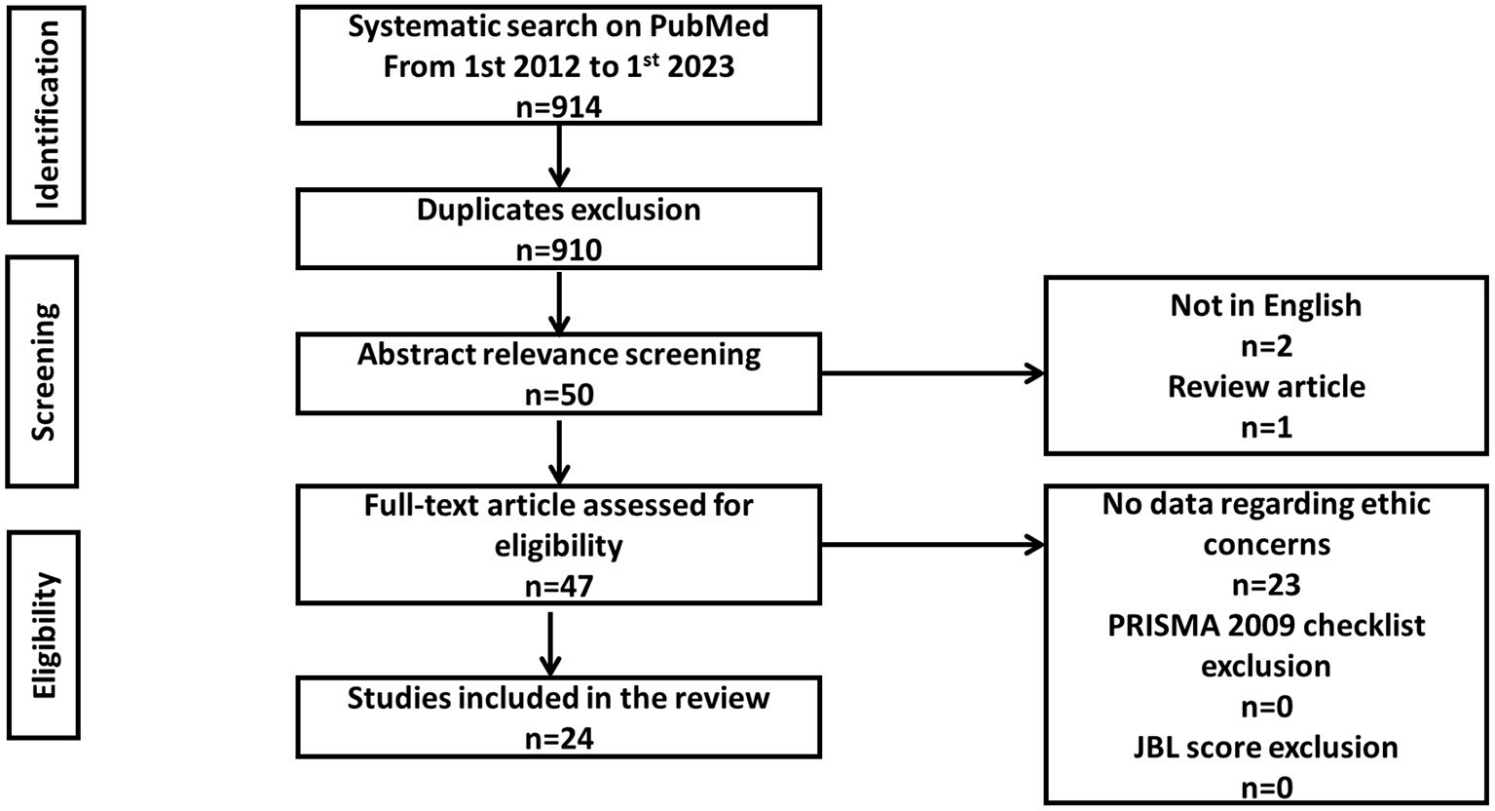
Flowchart for the exclusion criteria of the peer/reviewed papers.

A systematic search was conducted exclusively in the PubMed database, resulting in the identification of 914 articles based on the search query. Four (*n* = 4) articles were excluded as duplicates, resulting in 910 articles for further screening. The search query included the terms “Personalized medicine” to ensure comprehensive coverage of precision medicine-related literature since it is often used interchangeably. However, this broad search approach led to the inclusion of numerous irrelevant articles with “Personalized medicine” in their titles, after carefully screening of the titles and abstracts. The abstracts of the 910 articles, were screened for relevance by two independent authors. The screening of the abstracts, after application of inclusion and exclusion criteria, resulted in only fifty (*n* = 50) eligible articles to be further assessed in full text. Subsequently, two (*n* = 2) articles were excluded as they were not written in English and one (*n* = 1) article was identified as a review article (7), thus was excluded. However, all the references of the identified review article were also checked for eligibility. Full texts of the fifty articles underwent a subsequent quality assessment and assessment by two independent authors. From those fifty articles, only twenty-four (*n* = 24) were included in the analysis due to the availability of sufficient data on the topic. Articles that solely presented public opinion, expert opinion or lacked information on the risks or potential harms of precision medicine were excluded. The twenty-three excluded articles (*n* = 23) were evaluated as being out of the scope of this systematic review, since they did not include a sufficient quantitative or qualitative report of the participants on the subject. Finally, bias assessment of the individual studies was performed using the Joanna Briggs Institute (JBI) critical appraisal tool for analytical cross sectional, case control and case report studies, where applicable (11). Studies with fewer than five positive scores were excluded. The assessment is presented as Supplementary material. Thus, finally, 24 studies that report on the patients' perspectives regarding possible ethical concerns of precision medicine were included. Figure 1 shows the flowchart describing the inclusion/exclusion process. After study selection, the following data were extracted from full-text articles: eligibility criteria, study source and year, study design, country, sample size, age, gender and disease type.

All included studies were thematically analyzed for identifying themes related to ethical concerns and risks in precision medicine. The thematic analysis was performed on the relevant patients' opinion/perspectives regarding our question's topic. Data were retrieved from each study and classified through thematic analysis first in subthemes and subsequently into the different themes.

## Results

### Studies characteristics

The overall population sample size of the studies (*n* = 24) was 7,082 participants. These included 6,101 patients and 981 parents of patients. The majority of the studies included, were conducted in the USA (*n* = 17), while the other studies were conducted in Canada, England, Western Switzerland, France, Jordan, and Australia. The patients had been treated under diverse specialties such as Oncology, Rheumatology, Nephrology, Gastroenterology, Pulmonology and Primary Care, and had received a variety of services including genetic testing and pharmacogenetic. From the 24 studies, there were 7 studies of Primary Care patients, 5 studies of Oncology patients, 2 studies of Gastroenterology patients, 2 studies of Rheumatology patients, 1 study of Pulmonology and finally 1 study of Nephrology patients. Studies included are presented in Table 1.

**Table 1:** Studies included on patients' perspectives (*n* = 7,082) related to ethical issues and risks in precision medicine.

**Studies**	**Study type**	**Sample type and size**	**Participants age**	**Type of patients**	**Type of PM**	**Country**
Hassan et al. (12)	Qualitative	16 patients	16–18 & +18	Patients of the sheffield genetics service	Genomic medicine services	England
Gray et al. (13)	Qualitative	111 patients	32–86	Lung, breast, colorectal cancer	Somatic genetic testing	USA
Kraft et al. (14)	Qualitative	122 patients	20–95	Multiple specialty group practice	Not specified	USA
Issa et al. (15)	Quantitative	300 patients	18+	Breast and colorectal cancer	genomic diagnostics	USA
Woodbury et al. (16)	Qualitative	21 patients	Adult patients	Primary care	Not specified	USA
Beans et al. (17)	Qualitative	21 patients	Adult patients	Primary care	Not specified	USA
Ruel-Gagné et al. (18)	Quantitative	277 patients	50–65	Rheumatoid arthritis	Not specified	Canada
Chakravarthy et al. (19)	Quantitative	3847 patients	Adults	Academic medical centers, community-based hospitals, traditional outpatient clinics and federally qualified health centers	Not specified	USA
Williams et al. (20)	Quantitative	252 patients	Mean age 51.47	Primary care	Not specified	USA
Subasri et al. (21)	Qualitative	18 patients	35–84	Inflammatory bowel disease, gastrointestinal-related cancers	Pharmacogenomics,	USA
Sisk et al. (22)	Mixed method approach	804 parents of patients	Mean age 38.9	Pediatric Healthcare	Artificial Intelligence	USA
Schroll et al. (23)	Quantitative survey	252 patients	>21	Lung, Breast, Ovarian, Prostate, Bladder cancer	Not specified	USA
Puryear et al. (5)	Mixed method approach	100 patients	18+	Primary care	Not specified	USA
Perlman et al. (5)	Qualitative	34 patients	≥18	Syringe exchange program & HIV clinic	Genetic Testing and Genomic Medicine	USA
Boyer et al. (24)	Mixed method approach	10 patients	35–70	Primary care	Not specified	western Switzerland
Choukour et al. (25)	Qualitative	12 patients	average age 39.3	IBD patients	Not specified	France
Knoppers et al. (26)	Qualitative	22 patients	Adult patients	Cystic fibrosis	Not specified	Canada
Khdair et al. (27)	Quantitative	254 patients	18–70	Food and drug allergy. Hay-fever, asthma, eczema or urticaria. T1D, SLE, RA, MS, Psoriasis and Hashimoto	Not specified	Jordan
Hyams et al. (28)	Mixed-methods approach	17 patients	Mean 71.5 cases+69.7 control	Cancer	Genetic	USA
Cooke Bailey et al. (29)	Quantitative	103 patients	average 61.45	Chronic kidney disease	Not specified	USA
De Abreu Lourenco et al. (30)	Quantitative	130 parents of patients	25–74	Childhood cancer survivors	genomic medicine	Australian
Norstad et al. (31)	Mixed-methods approach	pediatric *n =* 32 and prenatal families *n =* 15	not indicated	Neurocognitive presentations or multiple congenital anomalies and pregnant women with undiagnosed fetal anomalies	exosome sequencing	USA
Lee et al. (14)	Qualitative	122 patients	20–95	Primary care	Not specified	USA
Diaz et al. (6)	Quantitative	190 patients	≥18 years	Primary care	Not specified	USA

### Patients' perspectives on ethics and potential risks regarding precision medicine

Through thematic analysis of the included studies, several ethical concerns and potential risks were identified and classified into eight main categories: privacy and security, economic impact, discrimination, informed consent, diagnostic accuracy, harms of precision medicine, mistrust in research and finally doctor-patient relationship. For detailed presentation of the themes per study please see Table 2.

**Table 2:** Identified Themes and subthemes of ethical issues and risks in precision medicine.

**Theme**	**Subthemes**	**Studies**
Privacy and security	Confidentiality	4, 5, 8, 10, 12, 13, 14, 17, 19, 22, 23
	Data sharing	1, 14, 20
	Management flow	1, 3, 5, 13, 14, 22
	Data security	1, 5, 11, 14
Economic impact	Cost	2, 6, 8, 10, 21, 24
	Loss of insurability	2, 6, 12, 18
	Loss of job	18
	Willingness to pay	4, 9
	Insurance coverage	11, 12
Discrimination		2, 3, 5, 12, 24
Informed consent		14, 17, 23, 24
Diagnostic accuracy	Accuracy of new developed tests	2, 4, 14
	Faith in technology	10
Harms of PM	Psychosocial harm	2, 18, 19
	Unexpected paternity	5
	Not taking care of yourself knowing your gene	5
	Human cloning	23
	Anxiety	8, 12, 14, 16
Mistrust in research	Trust in Provider	8
	Trust in Biomedical research	3, 7
	Trust in medical research	17
Doctor patient relationship		15

Hassan et al. (12); Gray et al. (13); Kraft et al. (14); Issa et al. (15); Woodbury et al. (16); Ruel-Gagné et al. (18); Chakravarthy et al. (19); Williams et al. (20); Subasri et al. (21); Sisk et al. (22); Schroll et al. (23); Puryear et al. (5); Beans et al. (17); Perlman et al. (5); Boyer et al. (24); Choukour et al. (25); Knoppers et al. (26); Khdair et al. (27); Hyams et al. (28); Hyams et al. (28); De Abreu Lourenco et al. (30); Norstad et al. (31); Lee et al. (14); Diaz et al. (6).

The most common theme identified was privacy and data security. Patients from a total of sixteen (*n* = 16) studies expressed worries regarding data security, confidentiality, reidentification, data management flow, data invasion by unauthorized parties (5, 12, 14–17, 20, 22, 23, 26, 28, 29, 31–33).

The second most common theme was the economic impact from the application of precision medicine, either the cost of the services of precision medicine or its impact by losing insurability. The economic impact was expressed in twelve (*n* = 12) studies, with six (*n* = 6) studies reporting on the cost of precision medicine (6, 13, 18, 20, 22, 30) and four (*n* = 4) studies reporting on the patients' concerns on losing illegibility for insurability (5, 13, 18, 27). In two studies, patients expressed their concern regarding the insurance coverage of tests of precision medicine (5, 23) whereas in two (*n* = 2) studies they reported their willingness to pay out of their own pocket for precision medicine applications (15, 21). Lastly, one (1) study in particular shows patients worry regarding the loss of their job due to precision medicine (27).

The third most common theme identified in nine (*n* = 9) studies was the possible harms of precision medicine including four (*n* = 4) studies reporting anxiety (16, 21, 22, 32), three (*n* = 3) studies reporting possible psychosocial harm (13, 27, 28), one study (*n* = 1) reported the risk of unexpected paternity, and not taking care of yourself due to your genes (16), as well as the risk of extra burden due to extra testing; and finally one (n=1) study expressed a fear of human cloning (31).

In five (*n* = 5) studies, patients reported concerns regarding discrimination due to their ethnic background (5, 6, 13, 16, 33). In particular, it was expressed that these concerns emerged from the historical mistreatment of certain races by medical professionals (33).

Another theme emerging from four (*n* = 4) studies was the concern regarding the process of informed consent and the usage of their data including their genetic information without the patients' consent (6, 14, 17, 26).

Furthermore, patients' confidence in their healthcare provider and trust in biomedical and medical research were significant factors that caused concern. This theme of mistrust in research was identified in four studies with the references (19, 20, 26, 33).

The seventh identified theme in three studies (*n* = 3) were the patients' concerns regarding the diagnostic accuracy of precision medicine. An example is recently developed tests such as next generation sequencing (13, 15, 17), while in one (*n* = 1) study, patients reported concerns about trusting and putting faith in the technology used (22).

Finally, there were concerns of the patients regarding the development of precision medicine leading to an impact on the doctor-patient relationship as they may rely more on the technology rather than on person follow ups (24).

### Perspectives of precision medicine among parents of patients

Among the studies three (*n* = 3) of the studies included reported on the parents' perspective (*n* = 981). The themes identified in these studies include concerns regarding economic impact of precision medicine such as cost of the tests of precision medicine (22, 30), re-identification and privacy risks for their children (31), the fear of losing their role in decision making as a result of using precision medicine tools with concerns regarding the quality and their faith in technology (22).

### Risk/benefits relationships of Precision Medicine approaches

Information on patients' perspectives on risk/benefits relationships of Precision Medicine approaches was lacking from the great majority of the studies included, and was only mentioned in three studies stating that from the patients' perspectives potential benefits overweigh the possible risks (5, 25, 33).

## Discussion

In this systematic review, we investigated the patients' perspectives toward the ethical issues and risks of precision medicine after screening 914 journal articles and finally including 24 articles. To our knowledge, this is the first systematic review that examines the perspectives of patients, identifying very few articles explicitly investigating the patients views toward the potential risks of precision medicine. The results of this review extend the current understanding of the application of precision medicine and the perspectives of patients regarding ethics and potential risks.

The most common theme identified was the patients' concern regarding privacy, confidentiality and security. Not surprisingly, privacy breach concerns were expressed between several different ethnic groups even though we live in an era where most of our life information is available online. While concerns regarding the security and privacy of the personal data were the most common, it should be taken into consideration that this could represent a bias due to the increasing attention on privacy issues, due to increased public awareness on the privacy of genetic data in particular, and the lack of extensive exploration of other ethical risks that precision medicine introduces (34). As precision medicine involves many types of data beyond genetic, privacy and confidentiality present an immense challenge and a major point of concern. In particular, personal data can be potentially used for commercial exploitation, as well as used as evidence against eligibility for insurance coverage or employment. Furthermore, patients that reported substance abuse, expressed concerns that their data could be potentially used to retract unsolved crimes and hence were reluctant to participate in precision medicine research programs (32). Interestingly, one of the studies demonstrated that the younger individuals are not so much concerned about privacy breach of their data but they were concerned about data accessibility (12). Finally, our study found that a portion of the patients are in favor of sharing their genomic data in a wider range but, as previously mentioned, this could be a subject of potential participation bias (12, 17, 29).

The second most common concern expressed by the patients was the economic impact of precision medicine which included the actual cost of the medical treatments and implications on insurance. The actual cost of the medical tests and its coverage by insurance companies as well as the loss of insurability due to having a genetic condition is a justified worry, which has led to the implementation of a new Law in the US called GINA which stands for Genetic Information Nondiscrimination Act (35). Other implications of the economic impact include insurance eligibility that might be jeopardized by findings of precision medicine and their ability to get employed (5, 13, 18, 27).

The theme regarding the possible harms of precision medicine included psychosocial harms and the need for genetic counseling. In particular, the potential increase in the number of diagnostic scans performed, can have a detrimental psychological impact on a patient that is already in distress where in extreme cases this psychological distress may lead to poorer prognosis (36). Research has shown that patients who undergo genetic testing and receive results indicating an increased risk for certain diseases may experience psychological distress and anxiety (34). This distress can have a negative impact on the patients' quality of life, as well as their ability to adhere to treatment and engage in healthy behaviors (35). Other more specific concerns include unexpected paternity and human cloning (14, 16). Finally, worrying about possible harms of precision medicine including “not taking care of yourself” while knowing your genes, is an issue that could improve through education. Especially regarding multifactorial diseases that can be influenced by genetics, epigenetics, environmental and lifestyle factors.

It is also essential to address concerns about unequal access to precision medicine and to ensure that patients from all backgrounds have access to these innovative treatments. From the patients' perspectives, concerns were raised regarding racial discrimination and the fear that their genes can be used against them. In particular, certain ethnic groups expressed mistrust that originated from historical evidence including the 1932 so called Tuskegee Syphilis Study which was a study that violated basic principles of bioethics that are autonomy, non-maleficence and injustice (33, 37).

There are several aspects regarding the patients' concerns on the process of informed consent. Firstly, patients expressed their worry about sharing their genetic information, through the usage of biospecimens especially genes and DNA, and possible future uses without their consent (6). In fact, hesitation on participating in genetic testing was evident, when the purpose of the genetic study was not clear, especially when patients were not fully informed of future uses without their consent (32). In addition, clear communication and the use of simple language on the consent form is important especially in clinical trials (26). However, patients' views can also vary, as some patients did not acknowledge the need of a different consent for their biospecimen for future research (14). Thus, the process of informed consent with clear communication following ethical guidelines, is critical to ensure that participants fully understand and consent to their involvement in research (36, 37).

Important concerns regarding trust in research were identified, especially when considering the origin of the studies and the ethnic background of the patients. In a study from Canada, patients showed trust to the researcher, with no hesitation to participate (26), in contrast to a study from the USA, in which Latinos showed mistrust in research mainly due to the unfamiliarity to the healthcare system (14). Similarly, other studies reported this diversity regarding trust in research influenced by the ethnic background of the patient (21, 38).

Concerns on diagnostic accuracy of newly developed genetic testing methods such as full genome sequencing were reported (11, 23). It seems that participants acknowledge the large quantity of possible uncertain results can lead to psychological distress and anxiety in patients, and a loss of trust in the medical system (17). In fact, a previous study supports these concerns, demonstrating how direct-to-consumer testing may be misleading when it comes to testing for familial hypercholesterolemia (39).

The implementation of precision medicine besides requiring the efficient collection and secure storage of huge amounts of data, also requires technological evolvement by means of Artificial Intelligence (AI) algorithms to process them. Regarding this technological evolvement, the patients' attitude is largely negative due to the fact that it could potentially deteriorate patient-doctor relationships as medical experts may rely more on the algorithms to predict outcomes in an effort to increase efficiency at the cost of their expertise.

Regarding, the unique group, including parents of patients (they were considered in the study as they are considered as a proxy for decision making on their children), concerns were expressed that these algorithms might have a negative effect on their decision making regarding their children's health and that this could eventually result in confusion and conflicts with medical experts. In contrast, their faith in technology was viewed as a positive attitude expressing their openness regarding AI.

It is important to mention that although a full analysis on risk/benefit relationships of precision medicine was not possible due to lack of data from the great majority of the original studies, the few studies reporting such data, supported the view that potential benefits overweigh the possible risks.

This study has many strengths. First, to our knowledge, it is the first systematic review that examines the perspectives of patients on ethics and potential risks related to the use of precision medicine. Second, the use of broad initial search criteria resulting in 914 possible articles, ensures that all the relevant literature was screened for inclusion. Moreover, the use of PRISMA guidelines for conducting a systematic review and the fact that all the steps of this study were made by two independent reviewers, reduces errors and possible biases. Some limitations of the present study should also be mentioned. First, the literature search was conducted in PubMed and in the English language only, thus possible bias cannot be excluded. Indeed, two publications, not written in English, were excluded. Also, the timeframe of the study, from 2012-−2023, although covering the years when applications in precision medicine expanded greatly, might have resulted in missing very early studies prior to the time period.

Finally, due to the limited qualitative data in the studies, the selection bias for the patients' perspective may be possible. Thus, although the thematic analysis in this review reached saturation, additional themes within the studies might have been neglected. In addition, in thematic saturation, when an observation does not contribute new themes, does not preclude a future observation from contributing new themes, thus further research might illuminate new aspects. Especially, as some themes were derived from a small number of studies, the present results should be interpreted with caution. Given the heterogeneity of the studies' design, a meta-analysis was not attainable. There was a great variation in the methodology applied in different studies including interviews, focus groups, questionnaires and surveys in different patient populations and settings. A bias toward patients from developed countries, especially USA was evident. Another issue is the fact that the majority of the included patients was recruited from primary care and secondly from specialized units including those suffering from rheumatological diseases and cancer. Thus, differences regarding the therapeutic area cannot be excluded and replication of the results is warranted. The lack of extensive standardized questionnaires and insufficient exploration of the patients' experience toward this rapidly evolving field, poses a potential risk of alienating medical experts and the public.

In conclusion, this study identified the main themes that emerge from the point of view of the patients regarding ethical issues and risks of precision medicine. These results give guidance on further actions that are needed to address these concerns. These include patient education and transparency on privacy issues, data protection and legal and economic concerns. Policies regarding insurability should also be a priority. In addition, issues on the effectiveness of precision medicine applications should be explained in detail to build trust and acquire the patients' informed consent. Educating patients about precision medicine is important to ensure that they are aware of the potential benefits and risks of genetic testing and targeted therapies. The implementation of a comprehensive educational program with written and online resources, incorporating both support groups and healthcare professionals taking into consideration the diverse backgrounds of the patients would be beneficial in the active involvement in precision medicine. Through empowerment, patients acquire knowledge and understanding about precision medicine, and they can consequently make informed decisions about their healthcare and advocate for their own interests. Psychological support should be offered when appropriate and physicians should be trained for clear patient communication to avoid miscommunication especially regarding complex tests, genetic counseling and precision medicine applications. Moreover, it is important to remind everyone involved, that the patient doctor relationship remains the cornerstone of practicing medicine and should not be compromised. Finally, more research is needed to identify present and forthcoming ethical issues and potential risks that may emerge from the implementation of precision medicine. Replication studies across diverse populations are necessary to assess the generalizability and consistency of findings.

## Data availability statement

The original contributions presented in the study are included in the article/Supplementary material, further inquiries can be directed to the corresponding author.

## Author contributions

LA participated in study design, data collection, and data analysis and drafted the manuscript. ACo participated in in study design and data analysis and data interpretation. ACh participated in study design, data analysis, interpretation of data, writing the manuscript and he was also the supervisor of LA, from which this manuscript was produced. All authors have read and approved the final manuscript.

## References

[1] Precision Medicine Initiative (PMI) Cohort Program [Internet]. NIMHD. Available online at: https://www.nimhd.nih.gov/programs/collab/pmi/ (accessed 1, March 2023).

[2] Leopold JA Loscalzo J. Emerging role of precision medicine in cardiovascular disease. Circ Res. (2018) 122:1302–15. 10.1161/CIRCRESAHA.117.310782 29700074 PMC6021027

[3] Bradley P Shiekh M Mehra V Vrbicky K Layle S Olson MC . Improved efficacy with targeted pharmacogenetic-guided treatment of patients with depression and anxiety: A randomized clinical trial demonstrating clinical utility. J Psychiatric Res. (2018) 96:100–7. 10.1016/j.jpsychires.2017.09.024 28992526

[4] National National Academies of Sciences Engineering and Medicine. Washington, DC: The National Academies Press. (2018).

[5] Puryear L Downs N Nevedal A Lewis ET Ormond KE Bregendahl M . Patient and provider perspectives on the development of personalized medicine: a mixed-methods approach. J Community Genet. (2018 J) 9:283–91. 10.1007/s12687-017-0349-x 29280052 PMC6002302

[6] Diaz VA Mainous AG Gavin JK Wilson D. Racial differences in attitudes toward personalized medicine. Public Health Genomics. (2014) 17:1–6. 10.1159/000354785 24080914

[7] Vetsch J Wakefield CE Warby M Tucker K Patterson P McGill BC . Cancer-related genetic testing and personalized medicine for adolescents: a narrative review of impact and understanding. J Adolesc Young Adult Oncol. (2018 J) 7:259–62. 10.1089/jayao.2017.0102 29336661

[8] Moher D Liberati A Tetzlaff J Altman DG PRISMA Group. Preferred reporting items for systematic reviews and meta-analyses: the PRISMA statement. Int J Surg Lond Engl. (2010) 8:336–41. 10.1016/j.ijsu.2010.02.007 20171303

[9] Jørgensen JT. Twenty years with personalized medicine: past, present, and future of individualized pharmacotherapy. Oncologist. (2019 J) 24:e432–40. 10.1634/theoncologist.2019-0054 30940745 PMC6656435

[10] Liberati A Altman DG Tetzlaff J Mulrow C Gotzsche PC Ioannidis JPA . The PRISMA statement for reporting systematic reviews and meta-analyses of studies that evaluate healthcare interventions: explanation and elaboration. BMJ. (2009) 339:b2700. 10.1136/bmj.b2700 19622552 PMC2714672

[11] Critical Appraisal Tools | JBI. Available online at: https://jbi.global/critical-appraisal-tools (accessed February 13, 2023).

[12] Hassan L Dalton A Hammond C Tully MP A. deliberative study of public attitudes towards sharing genomic data within NHS genomic medicine services in England. Public Underst Sci Bristol Engl. (2020) 29:702–17. 10.1177/0963662520942132 32664786 PMC7539600

[13] Gray SW Hicks-Courant K Lathan CS Garraway L Park ER Weeks JC. Attitudes of patients with cancer about personalized medicine and somatic genetic testing. J Oncol Pract. (2012) 8:329–35. 10.1200/JOP.2012.000626 23598841 PMC3500475

[14] Lee SSJ Cho MK Kraft SA Varsava N Gillespie K Ormond KE . “I don't want to be Henrietta Lacks”: diverse patient perspectives on donating biospecimens for precision medicine research. Genet Med Off J Am Coll Med Genet. (2019) 21:107–13. 10.1038/s41436-018-0032-6 29887604 PMC6289900

[15] Issa AM Tufail W Atehortua N McKeever J. A national study of breast and colorectal cancer patients' decision-making for novel personalized medicine genomic diagnostics. Pers Med. (2013 M) 10:245–56. 10.2217/pme.13.17 29768746

[16] Woodbury RB Beans JA Wark KA Spicer P Hiratsuka VY. Community perspectives on communicating about precision medicine in an Alaska native tribal health care system. Front Commun. (2020 S) 5:70. 10.3389/fcomm.2020.00070 33511166 PMC7839995

[17] Beans JA Woodbury RB Wark KA Hiratsuka VY Spicer P. Perspectives on precision medicine in a tribally managed primary care setting. AJOB Empir Bioeth. (2020) 11:246–56. 10.1080/23294515.2020.1817172 32940567 PMC7606746

[18] Ruel-Gagné S Simonyan D Légaré J Bessette L Fortin PR Lacaille D . Expectations and educational needs of rheumatologists, rheumatology fellows and patients in the field of precision medicine in Canada, a quantitative cross-sectional and descriptive study. BMC Rheumatol. (2021) 5:52. 10.1186/s41927-021-00222-2 34839831 PMC8627786

[19] Chakravarthy R Stallings SC Williams M Hollister M Davidson M Canedo J . Factors influencing precision medicine knowledge and attitudes. PLoS One. (2020) 15:e0234833. 10.1371/journal.pone.0234833 33175834 PMC7657499

[20] Williams JR Yeh VM Bruce MA Szetela C Ukoli F Wilkins CH . Precision medicine: familiarity, perceived health drivers, and genetic testing considerations across health literacy levels in a diverse sample. J Genet Couns. (2018) 3:291. 10.1007/s10897-018-0291-z 30105426 PMC6374217

[21] Subasri M Barrett D Sibalija J Bitacola L Kim RB. Pharmacogenomic-based personalized medicine: multistakeholder perspectives on implementational drivers and barriers in the Canadian healthcare system. Clin Transl Sci. (2021 N) 14:2231–41. 10.1111/cts.13083 34080317 PMC8604218

[22] Sisk BA Antes AL Burrous S DuBois JM. Parental attitudes toward artificial intelligence-driven precision medicine technologies in pediatric healthcare. Child Basel Switz. (2020) 7:145. 10.3390/children7090145 32962204 PMC7552627

[23] Schroll MM Agarwal A Foroughi O Kong E Perez O Pritchard D . Stakeholders perceptions of barriers to precision medicine adoption in the United States. J Pers Med. (2022) 12:1025. 10.3390/jpm12071025 35887521 PMC9316935

[24] Boyer MS Widmer D Cohidon C Desvergne B Cornuz J Guessous I . Representations of personalised medicine in family medicine: a qualitative analysis. BMC Prim Care. (2022) 23(1):37. 10.1186/s12875-022-01650-w 35232380 PMC8889694

[25] Choukour M Kivits J Baker A Baumann C Guillemin F Peyrin-Biroulet L. Personalised medicine in inflammatory bowel diseases: a patient survey. Scand J Gastroenterol. (2019 J) 54:135. 10.1080/00365521.2018.1555280 30663451

[26] Knoppers T Cosquer M Hagan J Nguyen MT Knoppers BM. “The stakes are higher”- patient and caregiver perspectives on cystic fibrosis research and personalized medicine. Front Med. (2022) 9:841887. 10.3389/fmed.2022.841887 35402437 PMC8984098

[27] Khdair SI Al-Qerem W Jarrar W. Knowledge and attitudes regarding genetic testing among Jordanians: an approach towards genomic medicine. Saudi J Biol Sci. (2021 J) 28:3989–99. 10.1016/j.sjbs.2021.04.004 34220256 PMC8241592

[28] Hyams T Bowen DJ Condit C Grossman J Fitzmaurice M Goodman D . Views of cohort study participants about returning research results in the context of precision medicine. Public Health Genomics. (2016) 19:269–75. 10.1159/000448277 27553645 PMC5053808

[29] Cooke Bailey JN Crawford DC Goldenberg A Slaven A Pencak J Schachere M . Willingness to participate in a national precision medicine cohort: attitudes of chronic kidney disease patients at a Cleveland public hospital. J Pers Med. (2018) 8:21. 10.3390/jpm8030021 29949895 PMC6164471

[30] De Abreu Lourenco R McCarthy MC McMillan LJ Sullivan M Gillam L. Understanding decisions to participate in genomic medicine in children's cancer care: a comparison of what influences parents, health care providers, and the general community. Pediatr Blood Cancer. (2021) 68:e29101. 10.1002/pbc.29101 34089211

[31] Norstad M Outram S Brown JEH Zamora AN Koenig BA Risch N . The difficulties of broad data sharing in genomic medicine: empirical evidence from diverse participants in prenatal and pediatric clinical genomics research. Genet Med Off J Am Coll Med Genet. (2022) 24:410–8. 10.1016/j.gim.2021.09.021 34906477 PMC12970631

[32] Perlman DC Gelpí-Acosta C Friedman SR Jordan AE Hagan H. Perceptions of genetic testing and genomic medicine among drug users. Int J Drug Policy. (2015) 26:100–6. 10.1016/j.drugpo.2014.06.013 25037119 PMC4276555

[33] Kraft SA Cho MK Gillespie K Halley M Varsava N Ormond KE . Beyond consent: building trusting relationships with diverse populations in precision medicine research. Am J Bioeth AJOB. (2018) 18:3–20. 10.1080/15265161.2018.1431322 29621457 PMC6173191

[34] Rahnama H Pentland A “Sandy,.” The New Rules of Data Privacy. Harvard Business Review. (2022). Available online at: https://hbr.org/2022/02/the-new-rules-of-data-privacy (accessed April 10, 2023).

[35] Rights (OCR) O for C. Genetic Information [Internet]. HHS.gov. (2008). Available online at: https://www.hhs.gov/hipaa/for-professionals/special-topics/genetic-information/index.html (accessed January 10, 2023).

[36] Custers JAE Davis L Messiou C Prins JB van der Graaf WTA. The patient perspective in the era of personalized medicine: what about scanxiety? Cancer Med. (2021 M) 10:2943–5. 10.1002/cam4.3889 33837668 PMC8085965

[37] Nix E. Tuskegee Experiment: The Infamous Syphilis Study. HISTORY. Available online eat: https://www.history.com/news/the-infamous-40-year-tuskegee-study (accessed January 10, 2023).

[38] Takashima K Maru Y Mori S Mano H Noda T Muto K. Ethical concerns on sharing genomic data including patients' family members. BMC Med Ethics. (2018) 19:61. 10.1186/s12910-018-0310-5 29914459 PMC6006763

[39] Sturm AC Truty R Callis TE Aguilar S Esplin ED Garcia S . Limited-variant screening vs. comprehensive genetic testing for familial hypercholesterolemia diagnosis. JAMA Cardiol. (2021) 6:902–9. 10.1001/jamacardio.2021.1301 34037665 PMC8156154

