# Supplementary material for: Patients' perspectives related to ethical issues and risks in precision medicine: a systematic review
Source: Front Med (Lausanne). 2023 Jun 15;10:1215663. doi: 10.3389/fmed.2023.1215663 (PMC10310545; doi:10.3389/fmed.2023.1215663)
Supplement: Supplementary file 1 [file Table_1.DOCX]

| **Studies** | | Q1 | Q2 | Q3 | Q4 | Q5 | Q6 | Q7 | Q8 | Q9 | Q10 | total |  |
| --- | --- | --- | --- | --- | --- | --- | --- | --- | --- | --- | --- | --- | --- |
| (Hassan et al., 2020) |  | 1 | 1 | 1 | 1 | 1 | 0 | 0 | 1 | 1 | 1 | 8 |  |
| (Gray et al., 2012) | | 1 | 1 | 1 | 1 | 1 | 0 | 0 | 1 | 0 | 1 | 7 |  |
| (Kraft et al., 2018) |  | 1 | 1 | 1 | 1 | 1 | 0 | 0 | 1 | 1 | 1 | 8 |  |
| (Issa et al., 2013) | | 1 | 1 | 1 | 1 | 1 | 0 | 0 | 1 | 1 | 1 | 8 |  |
| (Woodbury et al., 2020) | | 1 | 1 | 1 | 1 | 1 | 0 | 0 | 1 | 1 | 1 | 8 |  |
| (Beans et al., 2020) | | 1 | 1 | 1 | 1 | 1 | 0 | 0 | 1 | 1 | 1 | 8 |  |
| (Subasri et al., 2021) | | 1 | 1 | 1 | 1 | 1 | 0 | 0 | 1 | 0 | 1 | 7 |  |
| (Puryear et al., 2018) | | 1 | 1 | 1 | 1 | 1 | 0 | 0 | 1 | 1 | 1 | 8 |  |
| (Perlman et al., 2015) | | 1 | 1 | 1 | 1 | 1 | 0 | 0 | 1 | 1 | 1 | 8 |  |
| (Boyer et al., 2022) | | 1 | 1 | 1 | 1 | 1 | 0 | 0 | 1 | 1 | 1 | 8 |  |
| (Choukour et al., 2019) | | 1 | 1 | 1 | 1 | 1 | 0 | 1 | 0 | 0 | 0 | 6 |  |
| (Knoppers et al., 2022) | | 1 | 1 | 1 | 1 | 1 | 0 | 0 | 1 | 1 | 1 | 8 |  |
| (Hyams et al., 2016) | | 1 | 1 | 1 | 1 | 1 | 0 | 0 | 1 | 1 | 1 | 8 |  |
| (Cooke Bailey et al., 2018) | | 1 | 1 | 1 | 1 | 1 | 0 | 0 | 1 | 1 | 1 | 8 |  |
| (De Abreu Lourenco et al., 2021) | | 1 | 1 | 1 | 1 | 1 | 0 | 0 | 1 | 1 | 1 | 8 |  |
| (Norstad et al., 2022) | | 1 | 1 | 1 | 1 | 1 | 0 | 0 | 1 | 1 | 1 | 8 |  |
| (Lee et al., 2019) | | 1 | 1 | 1 | 1 | 1 | 0 | 0 | 1 | 1 | 1 | 8 |  |
| (Diaz et al., 2014) | | 1 | 1 | 1 | 1 | 1 | 0 | 0 | 1 | 1 | 1 | 8 |  |
| Q1. Is there congruity between the stated philosophical perspective and the research methodology?, Q2. Is there congruity between the research methodology and the research question or objectives?, Q3. Is there congruity between the research methodology and the methods used to collect data?, Q4. Is there congruity between the research methodology and the representation and analysis of data?, Q5. Is there congruity between the research methodology and the interpretation of results?, Q6. Is there a statement locating the researcher culturally or theoretically?, Q7. Is the influence of the researcher on the research, and vice- versa, addressed?, Q8. Are participants, and their voices, adequately represented?, Q9. Is the research ethical according to current criteria or, for recent studies, and is there evidence of ethical approval by an appropriate body?, Q10. Do the conclusions drawn in the research report flow from the analysis, or interpretation, of the data? © Joanna Briggs Institute 2017 Critical Appraisal Checklist for Qualitative Research (jbi.global) | | | | | | | | | | | | |  |
|  |  |  |  |  |  |  |  |  |  |  |  |  |  |
|  |  |  |  |  |  |  |  |  |  |  |  |  |  |
|  |  |  |  |  |  |  |  |  |  |  |  |  |  |
|  |  |  |  |  |  |  |  |  |  |  |  |  |  |

| **Studies** | Q1 | Q2 | Q3 | Q4 | Q5 | Q6 | Q7 | Q8 | total |
| --- | --- | --- | --- | --- | --- | --- | --- | --- | --- |
| (Ruel-Gagné et al., 2021) | 1 | 1 | 1 | 0 | 0 | 0 | 1 | 1 | 5 |
| (Chakravarthy et al., 2020) | 1 | 1 | 1 | 1 | 1 | 1 | 0 | 1 | 7 |
| (Williams et al., 2018) | 1 | 1 | 1 | 1 | 1 | 1 | 1 | 1 | 8 |
| (Sisk et al., 2020) | 1 | 1 | 1 | 1 | 0 | 0 | 1 | 1 | 6 |
| (Schroll et al., 2022) | 1 | 1 | 0 | 1 | 0 | 0 | 1 | 1 | 5 |
| (Khdair et al., 2021) | 1 | 1 | 1 | 1 | 0 | 0 | 1 | 1 | 6 |

Q1. Were the criteria for inclusion in the sample clearly defined, Q2. Were the study subjects and the setting described in detail?, Q3. Was the exposure measured in a valid and reliable way?, Q4. Were objective, standard criteria used for measurement of the condition?, Q5. Were confounding factors identified?, Q6. Were strategies to deal with confounding factors stated?, Q7. Were the outcomes measured in a valid and reliable way?, Q8. Was appropriate statistical analysis used? [© Joanna Briggs Institute 2017 Critical Appraisal Checklist for Analytical Cross Sectional Studies (jbi.global)](https://jbi.global/sites/default/files/2019-05/JBI_Critical_Appraisal-Checklist_for_Analytical_Cross_Sectional_Studies2017_0.pdf)
